# Factors Driving the Transition of Alzheimer’s Disease Patients to Institutional Long-term Care

**DOI:** 10.1093/geroni/igab046.2459

**Published:** 2021-12-17

**Authors:** Heather Davila, Guneet Jasuja, Lauren Moo, Madhuri Palnati, Qing Shao, Quanwu Zhang, Amir Abbas Tahami Monfared, Donald Miller

**Affiliations:** 1 Iowa City VA Healthcare System; University of Iowa Carver College of Medicine, Iowa City, Iowa, United States; 2 VA Bedford Healthcare System, Bedford, Massachusetts, United States; 3 VA Bedford Health Care System, Bedford, Massachusetts, United States; 4 Eisai Inc, Woodcliff Lake, New Jersey, United States; 5 Eisai Inc., Woodcliff Lake, New Jersey, United States

## Abstract

Progression of Alzheimer’s disease (AD) may ultimately lead to costly institutional long term care (ILTC) so its avoidance is often a goal of care management. We studied predictors of AD patients transitioning to ILTC in the Veterans Affairs healthcare system (VA). We identified 30,017 Veterans at least 50 years old, with ≥2 ICD-9/10-CM diagnosis codes for AD on separate days, with first AD code in 2013-2018, at least 2 years of prior continuous VA service use, and no prior ILTC. Patients who subsequently transitioned to ILTC (cases) were matched to other AD patients with the same time since first AD code but no ILTC (controls) (median of 13 months; mean age of 80.2 years). The 8,261 matched sets were split randomly to a training sample, where logistic and random forest regressions were used to develop models, and a validation sample, where final models were evaluated. Predictors of ILTC initiation included measures of (1) poor health, such as high morbidity counts (Elixhauser score of 15+, odds ratio=1.31) and weight loss (1.29), (2) heavy service use, such as hospitalization (2.25) and home health care (1.54), and (3) dementia symptoms, such as a diagnosis code for dementia not-otherwise-specified recorded well before the AD code (1.93), functional/mobility difficulties (1.35), and lifestyle or psychosocial problems (1.53). The full model C statistic was 0.78. Transition to ILTC in AD patients is driven by many factors, including comorbidities, need for acute care, nonspecific symptoms of dementias, and functional challenges. Targeted interventions may delay transitions to ILTC.

